# The Reversible Non-covalent Aggregation Into Fibers of PGLa and Magainin 2 Preserves Their Antimicrobial Activity and Synergism

**DOI:** 10.3389/fcimb.2020.526459

**Published:** 2020-09-30

**Authors:** Dennis Wilkens Juhl, Elise Glattard, Morane Lointier, Panos Bampilis, Burkhard Bechinger

**Affiliations:** ^1^University of Strasbourg/CNRS, UMR7177, Institut de Chimie de Strasbourg, Strasbourg, France; ^2^Institut Universitaire de France (IUF), Paris, France

**Keywords:** amyloid fiber, antimicrobial peptide, peptide-lipid interaction, supramolecular assembly, gradual release

## Abstract

Magainin 2 and PGLa are antimicrobial peptides found together in frog skin secretions. When added as a mixture they show an order of magnitude increase in antibacterial activity and in model membrane permeation assays. Here we demonstrate that both peptides can form fibers with beta-sheet/turn signature in ATR-FTIR- and CD-spectroscopic analyses, but with different morphologies in EM images. Whereas, fiber formation results in acute reduction of the antimicrobial activity of the individual peptides, the synergistic enhancement of activity remains for the equimolar mixture of PGLa and magainin 2 also after fibril formation. The biological significance and potential applications of such supramolecular aggregates are discussed.

## Introduction

Magainin 2 and PGLa were among the first cationic amphipathic peptides for which the biological regulation and antimicrobial activity was explored in greater depth (Zasloff, 1987; Gibson, 1991). In nature these peptides are both produced by and stored together in the granular glands of the South African clawed frog *Xenopus laevis* (Giovannini et al., 1987) and from each frog up to tens of milligrams of highly concentrated gel-like vesicular skin secretions have been isolated upon stimulation of release (Kiss and Michl, 1962; Gibson et al., 1986; Giovannini et al., 1987). Thereby the frogs have a potent innate defense mechanism that can be set in action immediately to prevent bacterial and fungal infections (Zasloff, 1987, 2002). Early on biophysical investigations provided experimental evidence that the cell killing by these and other cationic amphipathic peptides can be correlated with their selective impairment of bacterial plasma membrane function (Roversi et al., 2014; Aisenbrey et al., 2019). In contrast to the formation of barrel-stave pores observed for hydrophobic antimicrobial peptides such as alamethicin (Sansom, 1991; Salnikov et al., 2016) magainins and related linear cationic amphipathic sequences preferentially align parallel to the bilayer surface and work by different mechanisms (Bechinger et al., 1991; Oren and Shai, 1998; Bechinger, 2015).

Notably, equimolar mixtures made up of magainin 2a (GIGKF LHSAK KFGKA FVGEI MNS-NH_2_) and PGLa (GMASK AGAIA GKIAK VALKA L-NH_2_) have an almost one order of magnitude increased activity in antimicrobial assays but also in fluorescent dye-release studies from lipid-only model membranes (Westerhoff et al., 1995; Matsuzaki et al., 1998; Glattard et al., 2016). The molecular mechanisms for synergistic membrane disruption and antibacterial activities remains poorly understood and a number of models have been suggested (Matsuzaki et al., 1998; Marquette et al., 2015; Pino-Angeles et al., 2016; Zerweck et al., 2017; Leber et al., 2018). The biophysical investigations of their membrane interactions have recently been reviewed (Aisenbrey et al., 2019).

These biophysical studies show that magainin 2 adopts very stable alignments parallel to the membrane surface under all conditions tested so far (Bechinger, 2011; Salnikov and Bechinger, 2011; Strandberg et al., 2013), a feature also observed for many other related peptides (Porcelli et al., 2013; Resende et al., 2014; Perrin et al., 2015; Salnikov et al., 2018). In contrast to magainin 2, the membrane topological alignment of PGLa is modulated by the detailed composition of the phospholipid membrane, its peptide-to-lipid ratio, the hydration level or the presence of magainin 2 (Tremouilhac et al., 2006; Salnikov and Bechinger, 2011; Strandberg et al., 2013).

Whereas the interactions of antimicrobial peptides with lipids and other peptides within membranes have been the focus of prior biophysical investigations (Aisenbrey et al., 2019) much less is known about their interactions in solution. This is somewhat surprising because the potential of even very short polypeptides or extended amino acids to assemble into complex supramolecular assemblies has attracted increased attention in the context of biomaterials (Kumar et al., 2016; Chakraborty and Gazit, 2018; Beesley and Woolfson, 2019; Sun et al., 2019), biomedical applications (Fitzpatrick et al., 2013; Meier et al., 2014; Vermeer et al., 2017; Hainline et al., 2018) or amyloidogenic diseases (Fusco et al., 2016; Qiang et al., 2017; Ramamoorthy, 2018). Interestingly, the amyloid-beta peptide involved in Alzheimer disease has been found to not only assemble into fibrillar structures but to also exhibit antimicrobial activity (Soscia et al., 2010; Gosztyla et al., 2018). Fiber formation of some antimicrobial sequences has been described (Auvynet et al., 2008; Jang et al., 2011; Chu et al., 2012; Caillon et al., 2013) where some of the fibers are supramolecular arrangements made of peptide and acidic phospholipids (Sood et al., 2008; Mahalka and Kinnunen, 2009). In particular, magainin 2 has early on been published to aggregate into fibers at high salt and/or low pH conditions (Urrutia et al., 1989) but to our knowledge this observation has not been followed up in further investigations. Notably, amyloid fibers can have beneficial properties in helping with the long-term storage of antimicrobial peptides in the frog skin and thereby they constitute another example of functional amyloids that have previously been described (Maji et al., 2009; Depas and Chapman, 2012; Schubeis et al., 2015; Tayeb-Fligelman et al., 2017).

Therefore, in this work we have evaluated the fibrillation capacity of the antimicrobial peptides magainin 2a and PGLa as well as their synergistic equimolar mixture under physiological conditions. Moreover, we evaluated the antimicrobial activities of such fibrillar assemblies in comparison with non-aggregated peptides in solution. We have found that both peptides can form large supramolecular assemblies including fibers under physiological conditions and that this association has a strong effect on their antimicrobial activity. In order to evaluate structural details of the fibers biophysical techniques including EM-, FTIR-, and CD-spectroscopies are used and the results correlated to antimicrobial activity. Importantly, the peptides alone but also their synergistic mixtures have been investigated.

## Materials and Methods

### Peptide Synthesis

PGLa (GMASK AGAIA GKIAK VALKA L-NH_2_) and magainin 2a (GIGKF LHSAK KFGKA FVGEI MNS-NH_2;_ where the extension *a* is referring to the amidated carboxy terminus) were prepared by solid-phase synthesis using a Millipore 9050 automatic peptide synthesizer and Fmoc chemistry. The peptides were purified by reverse phase HPLC (Gilson, Villiers-le-bel, France) using a preparative C18 column (Luna, C-18-100Å-5 μm, Phenomenex, Le Pecq, France) and an acetonitrile/water gradient. Their identity and purity (>90%) were checked by MALDI-TOF mass spectrometry (MALDI-TOF Autoflex, Bruker Daltonics, Bremen Germany). The purified peptides were dissolved three times in 2 mM HCl at a 1 mg/mL concentration with subsequent lyophilization to ensure exchange of the TFA counter-ions. Quantification of peptides was based on mass and was determined for aliquots of ~10 mg peptide. Clean glass vials were weighted prior to the addition of the peptide solubilized in 2 mM HCl. The glass vials were weighted again after lyophilization. All vials were weighted 3 times before and after the addition of peptide on a 0.0001 g precision scale to account for small variations. Hereafter, the peptides were stored in aliquots of 1 mg at −20°C.

### Fibrillation in Tubes

Lyophilized powder of PGLa was dissolved in 50% ethanol at a 10 mM peptide concentration before being diluted into Mueller-Hinton (MH) medium or various buffers to a 0.5 mM final peptide concentration. All buffers were 50 mM and included: sodium phosphate at pH 7.4, sodium phosphate containing 150 mM NaCl at pH 7.4, sodium phosphate at pH 8.5, sodium phosphate at pH 3.5, Tris at pH 7.4; sodium acetate at pH 4.5; Hepes at pH 7.4, and ammonium bicarbonate at pH 7.4. Also, pure unbuffered Milli-Q filtered water was tested (Merck-Millipore, Molsheim, France). Fibrillation was conducted under vigorous shaking for 1 day.

### Determination of Fibrillation Yield

Fibrils prepared in tubes were centrifuged at 13,000 rpm for 30 min and the supernatants were removed. After lyophilization, the supernatants were dissolved in 2 mM HCl and analyzed by reverse phase HPLC (Bischoff chromatography, Leonberg, Germany) using an analytical C18 column (Luna, C-18-100 Å-5 μm, Phenomenex, Le Pecq, France) and an acetonitrile/water gradient. The flow rate is 1 ml/min, solvent A contains 10% acetonitrile in water, 0.1% TFA and solvent B is made of 100% acetonitrile, 0.1% TFA. The gradient for both peptides is 15% of solvent B for 3 min, an increase to 45% of solvent B within 15 min and finally to 95% for 3 min before the column is re-equilibrated with 15% B for 3 min. The peptide concentration was determined based on standard curves prepared from known peptide concentrations.

### Fibrillation in 96-Well Half Area Plates

The peptides were dissolved in 50% ethanol at a 10 mM peptide concentration before being diluted into MH medium or buffers containing 40 μM ThT. For each condition, 50 μL of the samples was transferred to three different wells in 96 half area well flat clear bottom black polystyrene TC-treated microplates (Corning, Corning Incorporated, Kennebunk, ME) before the fibrillation was carried out at 37°C with 300 rpm orbital shaking. The fluorescence of ThT was monitored on a CLARIO Star plate reader (BMG Labtech, Ortenberg, Germany) with 450 nm excitation and 485 nm emission filters. For seeded experiments, fibrils prepared in phosphate buffer by the tube method (cf. above), were vortexed vigorously and then added to each well such that the seeds correspond to 10% of the peptide concentration.

### Transmission Electron Microscopy

Sample material (10–20 μg) was deposited on formvar grids (CFT200-Cu, Electron Microscopy Sciences, Hatfield, PA, United States). After 2 min the grids were blotted dry and left to dry on air for another 30 min. The grids were washed twice in Milli-Q filtered water and a negative staining was made with 1% uranyl acetate in water for 1 min. TEM images were collected using a Hitachi H7500 electron microscope, operated at 80 KeV with a LaB6 filament.

### FTIR and Circular Dichroism Spectroscopy

The peptides were dissolved in 50% ethanol at a 10 mM peptide concentration before being diluted 1/20 into 1X MH medium or 50 mM phosphate buffer at pH 7.4. For peptide alone or mixture at molar ratio 1:1, the peptide solutions were transferred into 1.5 mL microtubes (V_f_ 500 μl; C_f_ 0.5 mM). Fibrillation was carried out with vigorous shaking in an Eppendorf Mixer 5432 for 1 day at room temperature. After centrifugation at 13,000 g for 30 min at RT, the pellets of the fibers were washed twice in 10 mM phosphate buffer, pH 7 in D_2_O.

For FTIR experiments, the pellets were resuspended with 10 μL of 10 mM phosphate buffer, pH 7 in D_2_O, of which 2 μL were loaded and dried on the diamond crystal. FTIR spectra (12 scans, from which the background was subtracted) were recorded from 1,500 to 2,500 cm^−1^ with a Nicolet 6700 spectrometer (Thermo Scientific, Waltham, MA).

For CD experiments, pellets from fibers were resuspended with 250 μL of 10 mM phosphate buffer, pH 7 and tip sonicated for 1 min at 50% power during 30% of the time (Sonoplus H200, Bandelin, Berlin, Germany). A second dilution by ½ is done if necessary. CD spectra (5–10 scans, from which a blank was subtracted) were recorded from 260 to 190 nm with a Jasco J-810 spectrometer (Jasco, Tokyo, Japan) using a 1 mm thick cuvette at 25°C.

### Antimicrobial Activity Assays

Fibrils of both peptides and their 1:1 mixture were prepared using the tube fibrillation in MH medium. Mueller Hinton medium (Fluka Analytical, Ref 70192, Lot BCBH7195V) contain beef infusion, casein hydrolysate and starch and it has a final pH of 7.3.

The fibrillation was initiated exactly 1 day before the antimicrobial activity assay. Similar samples were prepared half an hour before the assay. These last samples were not shaken. A gentle vortexing step was applied to all the samples prior to transferring to the microplate assuring a homogenous suspension of fibrils.

For all activity assays, *E. coli* bacteria (ATCC25922, Ref. 0335-CRM, Thermo Fisher Scientific, Courtaboeuf, France) were grown overnight on Mueller-Hinton agar plates. A suspension of bacteria in MH medium (Fluka Analytical, Sigma Aldrich, Saint-Louis, MO, USA) was made from the plates and used to inoculate a 10 mL preculture with a starting OD_550_ = 0.005. The preculture was incubated overnight and then used to inoculate a culture with a starting OD_550_ = 0.2 (10 mL of MH). The culture was incubated until an OD_550_ = 1.0 was reached (around 3 h). From this culture, a standard bacterial suspension was prepared with OD_550_ = 0.2 which were used to prepare the final bacterial working solution at OD_550_ = 0.0002.

The antimicrobial assays were performed in 96 well microplates (F-bottom sterile non-treated polystyrene, Thermo Scientific Nunc A/S, Roskilde, Denmark). All samples were submitted to the first column of the plate and subsequently exposed to a 2-fold dilution series in 10 steps. Finally, the bacterial working solution was distributed (50 μL) to each well except the blank controls. The final peptide concentration was ranging from 50 to 0.05 μM (after addition of bacteria).

The plates were incubated at 37°C for 18 h before the OD_600_ was measured. Resazurin was added to each well (0.033 mg/mL final dye concentration) and the plates incubated for another 2 h. The cell viability was determined based on the reduction of resazurin. The ratio of reduced resazurin was measured using the absorbance at 570 and 600 nm.

## Results

### PGLa and Magainin 2a Form Fibrils in Mueller Hinton Medium

The antimicrobial activities of magainin 2a and PGLa have been suggested to be associated with their cationic and amphiphilic character that allows them to partition into the interface of bacterial membranes (Bechinger, 1999; Zasloff, 2002). Later on, these and other α-helical peptides have been shown to assemble in mesophase arrangements along the membrane surface (Aisenbrey and Bechinger, 2014; Glattard et al., 2016; Marquette and Bechinger, 2018) or into fibrils (Urrutia et al., 1989; Soscia et al., 2010; Gosztyla et al., 2018) but the biological significance of such supramolecular assemblies still needs to be investigated. In this paper, we have studied the potential of magainin 2a and PGLa to form extended fibrillar structures under physiological conditions. Fibrillation assays were performed in Mueller Hinton Medium developed for antimicrobial assays or in buffers used in biochemical experiments. Furthermore, the antimicrobial activity of the resulting assemblies was tested and compared to that of non-aggregated peptides.

**Figure 1:**
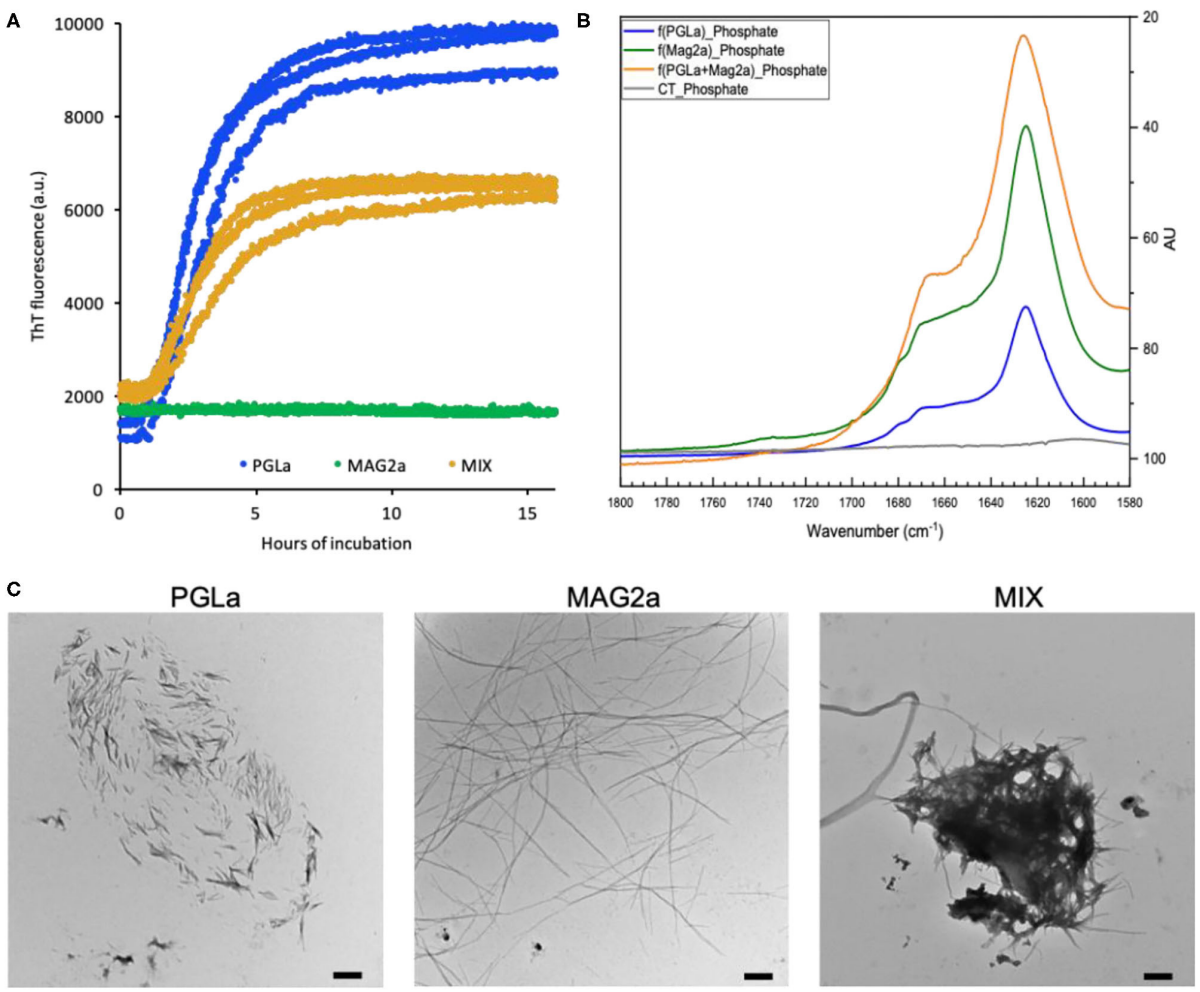
Fibril formation of PGLa, magainin 2 and their 1:1 mixture. **(A)** Fibrillation of 0.5 mM PGLa (blue), magainin 2 (green) and their 1:1 mixture (orange) in Mueller-Hinton medium monitored by ThT fluorescence. The fibrillation was conducted under constant shaking at 37°C. **(B)** FTIR spectra of fibrils prepared from PGLa (blue), magainin 2 (green) and their 1:1 mixture (orange) in 50 mM phosphate buffer, pH 7.4. The buffer alone is shown in gray. **(C)** TEM images of fibrils prepared from PGLa, magainin 2 and their 1:1 mixture in Mueller-Hinton medium. The scale bars indicate 500 nm.

Fibrillation was conducted at a 1 mg/mL peptide concentration (0.5 mM) in microplates under continued shaking. The amyloidogenic dye thioflavin T was included in the samples as a reporter for fibrillar structures. After ~1 h, an increase in thioflavin T fluorescence was observed for both PGLa and an equimolar mixture of PGLa and magainin 2 (Figure 1A). The fluorescence continued to increase until a plateau was reached after ~8 h. After normalizing to the final fluorescence level, the two curves overlap (not shown). The similar fibrillation kinetics indicates that PGLa fibrillation is independent of the presence of magainin 2a. For magainin 2a alone, no increase in the ThT fluorescence was observed during the 16 h duration of the experiment.

The structures formed during the fibrillation assay were evaluated by electron microscopy (EM). Despite the lack of ThT fluorescence in the case of magainin 2a, all three samples exhibit assemblies of fibrillar structures (Figure 1C). Even though some inhomogeneity in the fibril morphologies was observed by EM, the fibrils generated by PGLa could be distinguished from those generated by magainin 2a. On the EM grids, PGLa appears as larger assemblies of short rigid fibril sticks while magainin 2a forms long thin fibrils, which did not seem to assemble further. The EM images of the peptide mixture appear to contain both types of fibrillar assemblies suggesting that the two peptides de-mix when forming fibrils. This is supported by the similar fibrillation kinetics observed for PGLa alone and in the peptide mixture (Figure 1A).

Thioflavin T becomes fluorescent when interacting with the repetitive sidechain arrangements on the surface of some β-sheet amyloidogenic structures (Biancalana and Koide, 2010). However, the intensity of the fluorescent signal varies substantially depending on the exact fibrillar surface defined by the peptide sequence and fibril morphology (Juhl et al., 2019). Using FTIR spectroscopy of fibrils prepared in phosphate buffer, we confirm secondary structures closely resembling those of other amyloids (Shivu et al., 2013) as the spectra are dominated by intensities around 1,623 cm^−1^ for all three samples (Figure 1B). Spectral deconvolution is indicative of 75–60% β-sheet/β-turn structures and ca. 20% helical conformations (not shown) (Goormaghtigh et al., 1990). CD-spectra of sonicated fibers are characterized by minima around 215 nm thus confirming the preference for β-conformations, while a quantitative analysis is hampered by light scattering artifacts (Supplementary Figure 1).

### Mixed Fibrils Retain the Activity in Contrast to Fibrils of the Individual Peptides

In a next step, the effect of fibril formation was assayed by comparing the antimicrobial activity of freshly prepared peptide solutions with those from samples exposed to continuous shaking for 24 h. The antimicrobial activity assay was conducted as previously reported (Glattard et al., 2016) with the final peptide concentration ranging from 50 to 0.05 μM in a 2-fold dilution series encompassing 10 steps. The fibrils and peptides eventually remaining in solution after 24 h of incubation were not separated from each other for the antimicrobial activity test.

**Figure 2:**
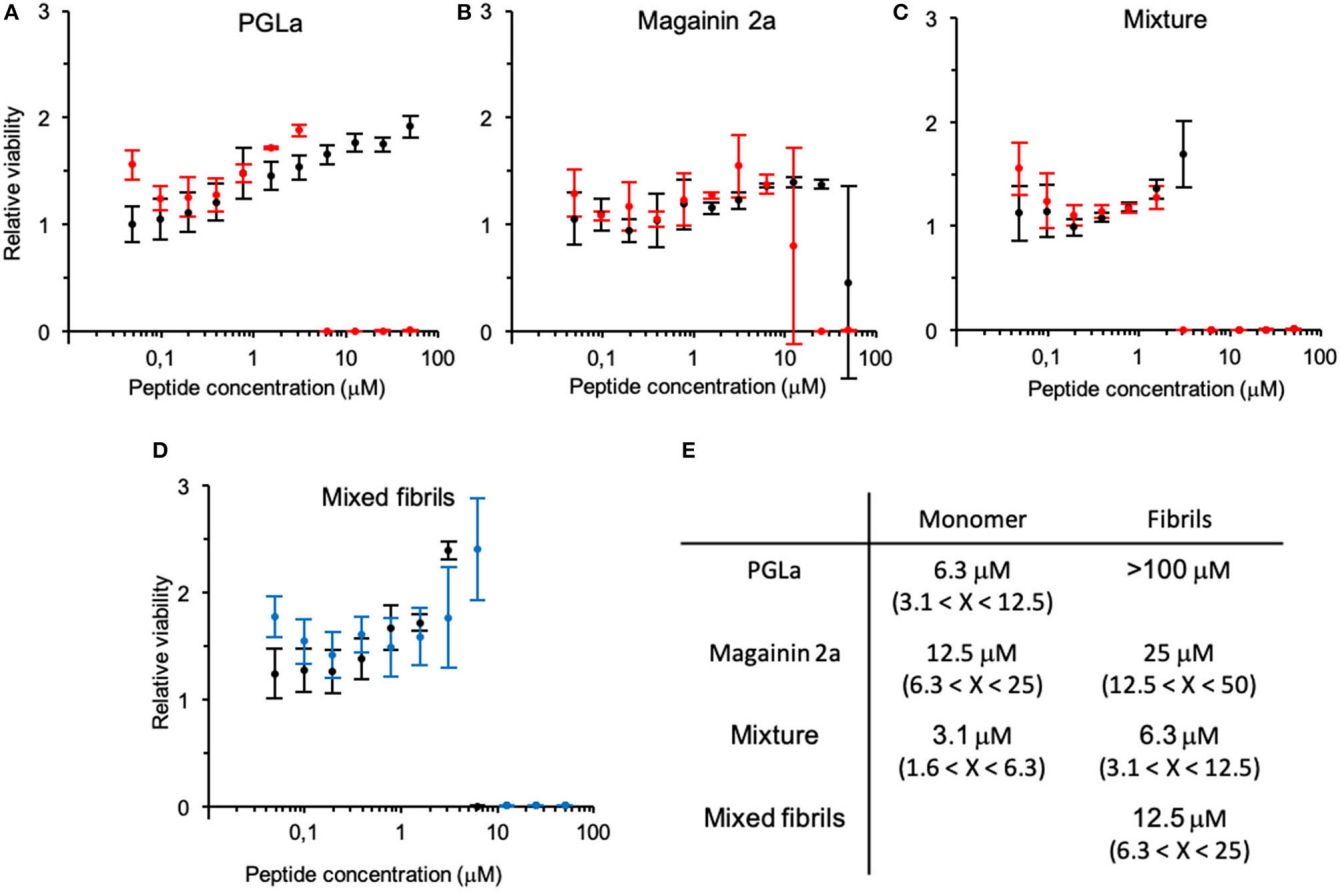
Change in antimicrobial activity upon peptide fibrillation. The cell viability of bacteria incubated with fibrillated (black) or monomeric (red) peptide solutions relative to bacteria grown alone is depicted at increasing concentrations of **(A)** PGLa, **(B)** magainin 2 and **(C)** their 1:1 mixture (top panel). **(D)** Antimicrobial activity assay of the 1:1 mixture of PGLa and magainin 2 mixed before (black) or after (blue) fibrillation (bottom panel). Note, the error bar at the steep transition at the MIC reflects contributions from the horizontal and vertical axis. **(E)** Table summarizing the MIC. Antimicrobial activity was tested against *E. coli* (ATCC25922). Errors arise from the 2-fold peptide dilution steps of the assay.

Formation of fibrils leads to a decrease in antimicrobial activity (Figure 2). In particular, the PGLa fibril formation completely inhibited the activity of the peptide (Figure 2A). The minimal inhibitory concentration (MIC100) of magainin 2a changed from around 15 μM before fibrillation to around 30 μM after fibrillation (Figure 2B), while the MIC100 of the 1:1 peptide mixture doubled from 3 to 6 μM (Figure 2C). Finally, we assayed the activity of a 1:1 mixture of PGLa fibrils and magainin 2a fibrils when the peptides were mixed after fibrillation (Figure 2D). Here the activity dropped slightly (MIC100 13 μM) when compared to mixtures fibrillated together, but still showed increased activities when compared to the individual peptides.

Measurements of antimicrobial activity during the time course of the fibrillation process were performed by taking samples at 30 min, 1, 4, and 24 h. These measurements confirmed the trends of the previous experiments. For PGLa, the activity dropped already after 1 h of shaking and disappeared completely after 4 h. For magainin 2a, the changes appeared at an intermediate time scale, while the peptide mixture only showed changes after 24 h of incubation (data not shown).

### Searching the Mechanism Behind PGLa Fibrillation

**Figure 3:**
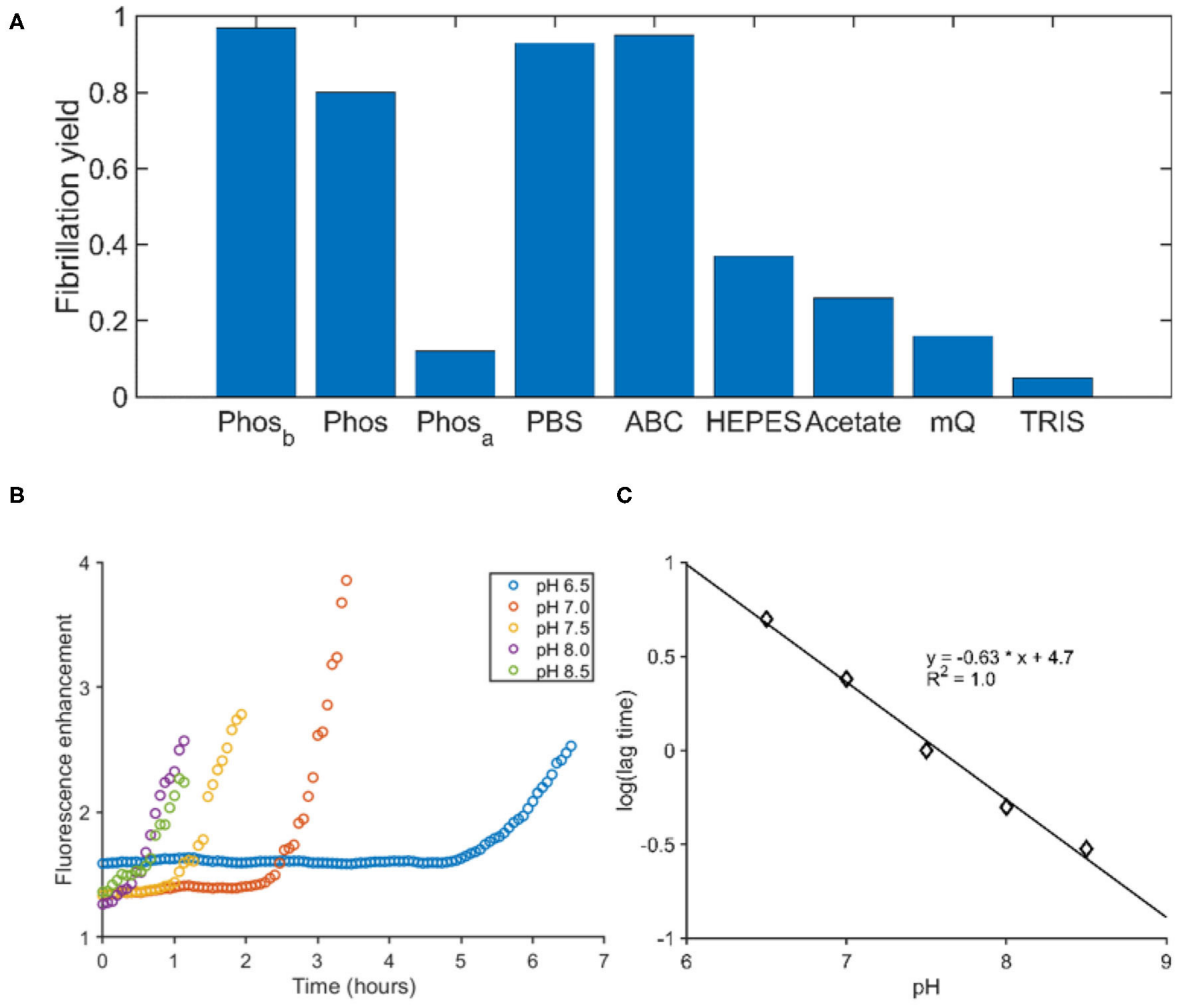
The influence of buffer composition on the fibrillation of PGLa. **(A)** The fibrillation yield of PGLa was determined by HLPC after 24 h of continuous shaking in various buffers. All buffers were at a 50 mM concentration. Phosphate (Phos), PBS, ammonium bicarbonate (ABC), HEPES and TRIS were adjusted to pH 7.4, while the acetate buffer was adjusted to pH 4.5. Phos_b_ and Phos_a_ were prepared like the phosphate buffer and thereafter the pH was changed outside of the buffer regime by addition of either base (Phos_b_) or acid (Phos_a_), respectively. **(B)** The fibrillation kinetics of PGLa was followed by ThT in phosphate buffers as a function of pH. **(C)** The pH of the phosphate buffer correlates linearly to the logarithm of the lag time.

To identify factors influencing fibril formation, we determined the yield of aggregation for both peptides in different buffers. The aggregation was performed in tubes and the yield was determined by quantifying the peptide in the supernatant after centrifugation. For both peptides, phosphorous ions facilitated the aggregation, while PGLa also aggregated in the presence of ammonium bicarbonate (Figure 3A). The buffers TRIS, HEPES and acetate at pH 7.4 as well as pure water did not promote aggregation within 24 h and the addition of 150 mM sodium chloride to the phosphate buffer had only little consequences. In all instances of significant aggregation, EM pictures revealed fibrillar assemblies (Supplementary Figure 2). Finally, the ThT fluorescence of PGLa fibrils was exploited to follow the fibrillation in phosphate buffers at different pH. By varying the pH from 6.5 to 8.5, a shortening of the lag time was observed when the pH was increased (Figure 3B). Plotting the logarithm of the lag time against the pH gives a linear correlation (Figure 3C).

When preformed PGLa fibrils prepared in phosphate were suspended into other buffers without phosphate, the fibrils disassociated (data not shown). Likewise, lowering the pH below the buffer range of phosphate (i.e., pH ≈ 3) results in the dissolution of the fibrils. The only exception was 50% ethanol which might be too hydrophobic to solvate the phosphate and lysine ions. Furthermore, when the concentration of non-aggregated peptide was tested by separating the fibers from the solution by centrifugation the supernatant contained only 10–20% of the initial peptide in phosphate buffer but a much higher fraction remained in Tris buffer solution (not shown).

## Discussion

Here we demonstrate that the antimicrobial peptides magainin 2a and PGLa are prone to form fibrous molecular aggregates in the presence of bivalent anions at physiological conditions. These fibers can reversibly dissociate when exposed to aqueous environments of appropriate composition (e.g., acidic conditions, absence of phosphate or presence of detergents). The peptides investigated here are produced and stored together at high concentrations in the granular glands of the *Xenopus laevis* frog skin (Giovannini et al., 1987). Both peptides exhibit antimicrobial activities against a broad range of Gram-negative or Gram-positive bacteria and fungi (Soravia et al., 1988; Zasloff et al., 1988). They are released when the amphibian skin is stimulated or when exposed to bacterial infection (Giovannini et al., 1987). The supramolecular aggregate may thus help in efficient storage, protect these peptides from proteolytic degradation (Giovannini et al., 1987) and allow for their slow release over extended time periods similar to the storage of peptides and peptide hormones of the mammalian endocrine system (Maji et al., 2009). Thus, the peptides form a functional fiber beneficial to the producing organisms similar to other functional amyloids. For example, functional amyloids are formed by the phenol-soluble modulins of *S. aureus*, which constitute a virulence factor and help in biofilm formation of this bacterium (Tayeb-Fligelman et al., 2017).

Charges are well-known to influence the fibrillation kinetics of peptides and proteins (Zapadka et al., 2017). The charges can be soluble ions like in our study, associated with the surface of the container or be an additional fibrillation partner like Heparin sulfate (Risor et al., 2017). In all cases, the negative charges screen positive side-chains which reduces electrostatic repulsion and/or bridges cationic sites. At the same time, the arrangement of negative charges might bring the proteins closer to each other thereby improve their propensity for aggregation.

Magainin 2a and PGLa contain four lysines and a free amino terminus which, at neutral pH, nominally result in an overall positive net charge of 3–4 and 4–5, respectively. The high cationic character keeps the peptides in solution and enhances the interaction with the anionic surfaces of bacterial membranes (Matsuzaki et al., 1995; Bechinger, 1999). However, both peptides also contain a significant number of residues which confer considerable hydrophobic character driving aggregation (Torrent et al., 2011a,b; John et al., 2019). Notably, for other cationic amphipathic peptides amyloid formation has been postulated to pass through a loosely packed helical intermediate (John et al., 2019). Therefore, it is worth mentioning that both peptides can adopt amphipathic helical structures with high hydrophobic moment and a clear separation of charged/polar and hydrophobic residues (Marquette et al., 2015).

As PGLa does not contain amino acid side chains with a pK in the physiological pH range, the pH-dependent lag time and fibrillation kinetics probably mirrors the phosphate protonation state as was previously observed for another cationic peptide (Vermeer et al., 2017). For the pH range 6.5–8.5 the H_2_${\text{PO}}_{3}^{-}$ and ${\text{HPO}}_{3}^{2-}$ ions of the buffer are in equilibrium, where the divalent anion increases in concentration in a logarithmic fashion with pH. Furthermore, the amino terminus of polypeptides is typically characterized by a pK value close to the upper pH range investigated here and its deprotonation may help in the fiber assembly. Together, pH increase results in an effective reduction in peptide charge and the screening of remaining charges by phosphate ions thereby promoting the onset of fibril formation.

We show here, that divalent phosphate ions can induce fibrillation for both peptides, while carbonate works for PGLa as well (Figure 3A). The stability of the fibrils is dependent on the presence of those ions, possibly due to a cross-linking of positively charged lysine side chains from opposing peptide chains.

Fibrillation of the antimicrobial peptides results in a moderate (magainin 2a) to considerable loss in antimicrobial activities (PGLa). The reduced activity of the fibers can be explained by a number of factors. First, one can expect that the fibers face difficulties to pass the lipopolysaccharide layer, the outer membrane and peptidoglycan structures of the bacteria. Second, the β-sheet amyloids of these antimicrobial peptides form by interacting with bivalent anions. Thereby, some of the cationic charges of the peptide side chains are screened and the net charge per polymeric units is reduced. This should result in a lower local polypeptide concentration next to the plasma membrane surface (Wieprecht et al., 2000). Third, the membrane partitioning of such large complexes may be more difficult to achieve.

Based on the fibrillation yields, the activity should have decreased even further if the fibrils were completely inactive and stable. Possibly, the fibrils retain some activity on their own as has been shown for Aβ-fibers which exhibit antimicrobial action. It has been suggested that the latter are capable to interact with membranes and to trap infectious bacteria (Chairatana and Nolan, 2014; Gosztyla et al., 2018). By flocculation and sequestration the spread of pathogens is limited and they are prepared for phagocytosis (Robinson and Bishop, 2002).

The peptides' ability to re-dissolve presents another possibility on how antimicrobial activity is restored. Thus, competition between the anionic lipids and the bivalent anions that are required to glue the fibers together can be a driving force for the dissociation of the fibers. Along the same line, the multiple cationic charges of antimicrobial peptides have been suggested to compete with the bivalent cations that are required to electrostatically cross-link the bacterial lipopolysaccharide network (Hancock, 1984). Thereby the addition of bacteria to the sample might be sufficient to shift the equilibria from fibers to soluble peptide to peptides associated with the bacterial surfaces (Savini et al., 2017). The reversibility of the fibril formation could also explain the pronounced activity of the peptide mixture.

The peptides investigated here show antimicrobial activities in the 10 μM range as observed previously (Zasloff, 1987; Bevins and Zasloff, 1990), a value that drops considerably when both peptides are mixed at an equimolar ratio (Juretic et al., 1989; Matsuzaki et al., 1998; Glattard et al., 2016). The underlying reason for this synergistic enhancement remains a matter of research (Matsuzaki et al., 1998; Salnikov and Bechinger, 2011; Strandberg et al., 2013; Zerweck et al., 2017; Harmouche and Bechinger, 2018; Leber et al., 2018; Aisenbrey et al., 2019). When the fibers are tested, this synergistic enhancement is maintained. Although this synergistic effect cannot be quoted in a truly quantitative manner due to the complete lack of antimicrobial activity of the PGLa fibers at up to 50 μM concentrations, the mixed fiber retains half of the activity when compared to the non-aggregated peptide mixtures. Furthermore, a mixture of the independently prepared fibers retains considerable activity. Also, in this case the peptides can dissociate from the fiber and insert into the membrane. It has been suggested that magainin 2 and PGLa weakly interact with each other by forming parallel helical arrangements (Matsuzaki et al., 1998; Marquette and Bechinger, 2018). Although the β-sheet structure and the α-helical arrangement in membranes are different from each other it is possible that the fiber dissociates into small oligomers/dimers which may interact directly with the membrane whereby they undergo a structural transition. When added in their monomeric forms PGLa has been suggested to precondition the membrane for magainin, which then exerts its optimal activity (Matsuzaki et al., 1998; Leber et al., 2018). In an analogous manner, the presence of magainin could help PGLa to be in a more active supramolecular arrangement when encountering the bacterial membrane e.g., by dissociating into small active oligomers more easily to cite just one of many possible explanations. In this context it is worth mentioning that the 17-residue uperin 3.5, another peptide from frogs, has been shown to exist as random coil monomers, a lose helical intermediate from which fibers can form in the presence of phosphate buffer (Calabrese et al., 2016; John et al., 2019), all of which can interact with membranes (Martin et al., 2018).

So far, magainin and PGLa are thought to exert their antimicrobial and synergistic activities by partitioning into the membrane interface where they adopt amphipathic helical secondary structure (Marquette and Bechinger, 2018; Aisenbrey et al., 2019). Helices in such interfacial situations result in considerable curvature strain (Harmouche and Bechinger, 2018), but membranes can adapt until higher peptide densities cause local phase changes allowing the transient passage of ions and water (SMART model, Bechinger, 2015). Furthermore, mesophase arrangements along the membrane surface have been measured for amphipathic cationic peptide helices including magainin, PGLa and their mixtures (Aisenbrey and Bechinger, 2014; Glattard et al., 2016; Marquette and Bechinger, 2018). For mixtures of magainin 2 and PGLa parallel heterodimers have been observed (Hara et al., 2001) which can potentially enhance the membrane disruptive activities of the individual components (illustrated in Bechinger, 1999; Aisenbrey et al., 2019). Whereas, under the conditions of this study fiber formation made from β-sheets/turns has been observed in aqueous solution for PGLa and magainin 2a (Figure 1), in the presence of membranes the supramolecular ordered arrangements of the peptide-lipid complexes have been shown to encompass helical secondary structures (Matsuzaki et al., 1998; Aisenbrey et al., 2019). While for other cationic amphipathic peptide amyloid formation has been postulated to pass a loosely packed helical intermediate (John et al., 2019) future research is required to investigate the reverse process. In particular, the β-sheet fibrils in aqueous solution are capable to interact with membranes and to convert cooperatively into a helical assembly, or they have to dissociate first before interacting with lipid bilayers.

In summary, the antimicrobial peptides PGLa and magainin 2a form fibrillar aggregates in physiological aqueous solutions. Their antimicrobial activities decrease with fibril formation, but do not disappear completely. Mixing the peptides, either monomeric or in fibrillar form, results in an increased activity with little effect of the formation of fibrils. The antimicrobial activity of mixed fibrils of PGLa and magainin 2a can potentially be exploited for different future applications. Structurally, the fibrils are more resistant toward proteases and therefore constitute a possible state for long-term storage as may be the case in the granular glands. Moreover, the rapid switch to an active state upon addition of bacteria makes the fibrils ideal for surface coating of material where bacterial growth would be fatal. Finally, fibers made from peptides of similar size have been found key elements to enhance lentiviral transduction in clinical settings (Meier et al., 2014; Majdoul et al., 2016; Vermeer et al., 2017), and a number of other peptides may be suitable for the targeted delivery of macromolecules and biomolecular assemblies (Moulay et al., 2017).

## Data Availability Statement

The datasets generated for this study are available on request to the corresponding author.

## Author Contributions

DJ, EG, ML, and PB did the experiments. BB and DJ wrote the paper. BB acquired funding for the project. All authors contributed to the article and approved the submitted version.

### Conflict of Interest

The authors declare that the research was conducted in the absence of any commercial or financial relationships that could be construed as a potential conflict of interest.

## Abbreviations

ABC: ammonium bicarbonate
CD: circular dichroism
*E. coli*: *Escherichia coli*
EM: electron microscopy
FRET: fluorescence resonance energy transfer
FTIR: Fourier transform infrared
HCl: hydrochloric acid
HEPES: 4-(2-Hydroxyethyl)piperazine-1-ethanesulfonic acid
MALDI-TOF: matrix-assisted laser desorption ionization-time of flight
MIC: minimal inhibitory concentration
NMR: nuclear magnetic resonance
OD: optical density
PGLa: peptide starting with a glycine and ending with a leucine amide
HPLC: high performance liquid chromatography
RT: room temperature
TEM: transmission electron microscopy
TFA: trifluoroacetic acid
ThT: thioflavin T
Tris, 2-amino-2-(hydroxymethyl)-1: 3-propanediol.
