# Supplementary material for: The Reversible Non-covalent Aggregation Into Fibers of PGLa and Magainin 2 Preserves Their Antimicrobial Activity and Synergism
Source: Front Cell Infect Microbiol. 2020 Sep 30;10:526459. doi: 10.3389/fcimb.2020.526459 (PMC7554302; doi:10.3389/fcimb.2020.526459)
Supplement: Supplementary file 1 [file Data_Sheet_1.pdf]

Supplementary Information

The Reversible Non-covalent Aggregation Into Fibers of PGLa and Magainin 2 Preserves Their Antimicrobial Activity and Synergism

Dennis Wilkens Juhl[1,†], Elise Glattard[1], Morane Lointier[1], Panos Bampilis[1], Burkhard Bechinger[1, 2,*]

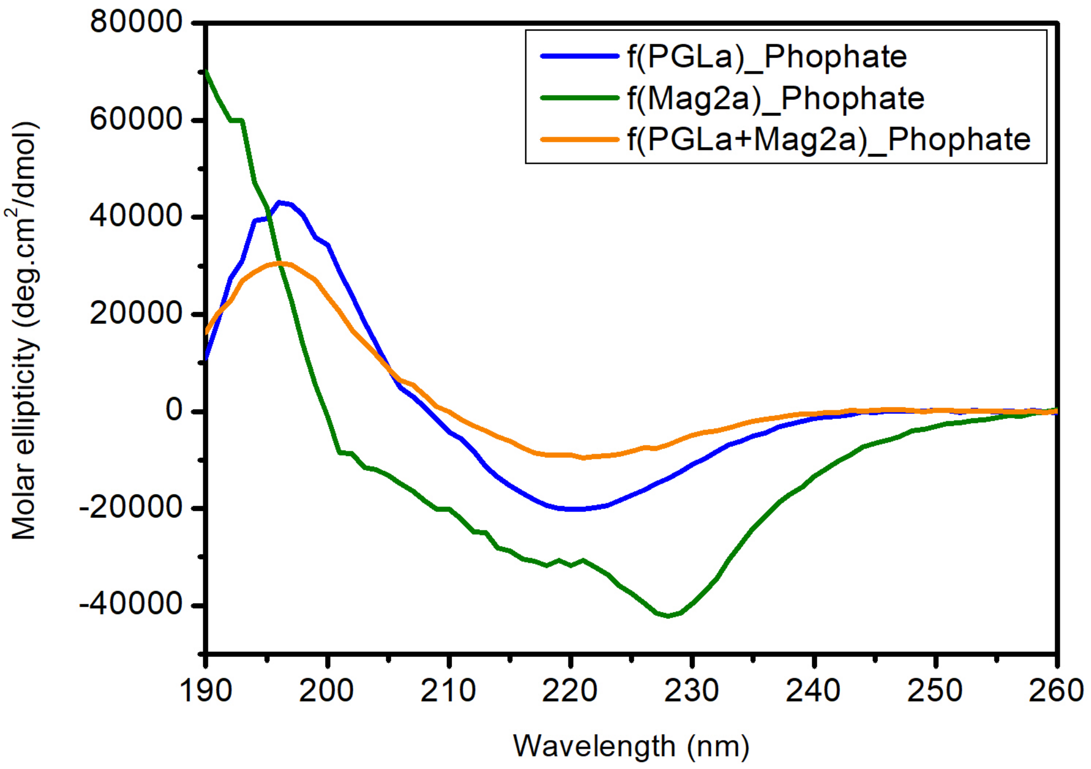

Figure S1: CD spectra of fibers made of PGLa, magainin and an equimolar peptide mixture in phosphate buffer. Because of strong aggregation artifacts the spectra were not analyzed further.

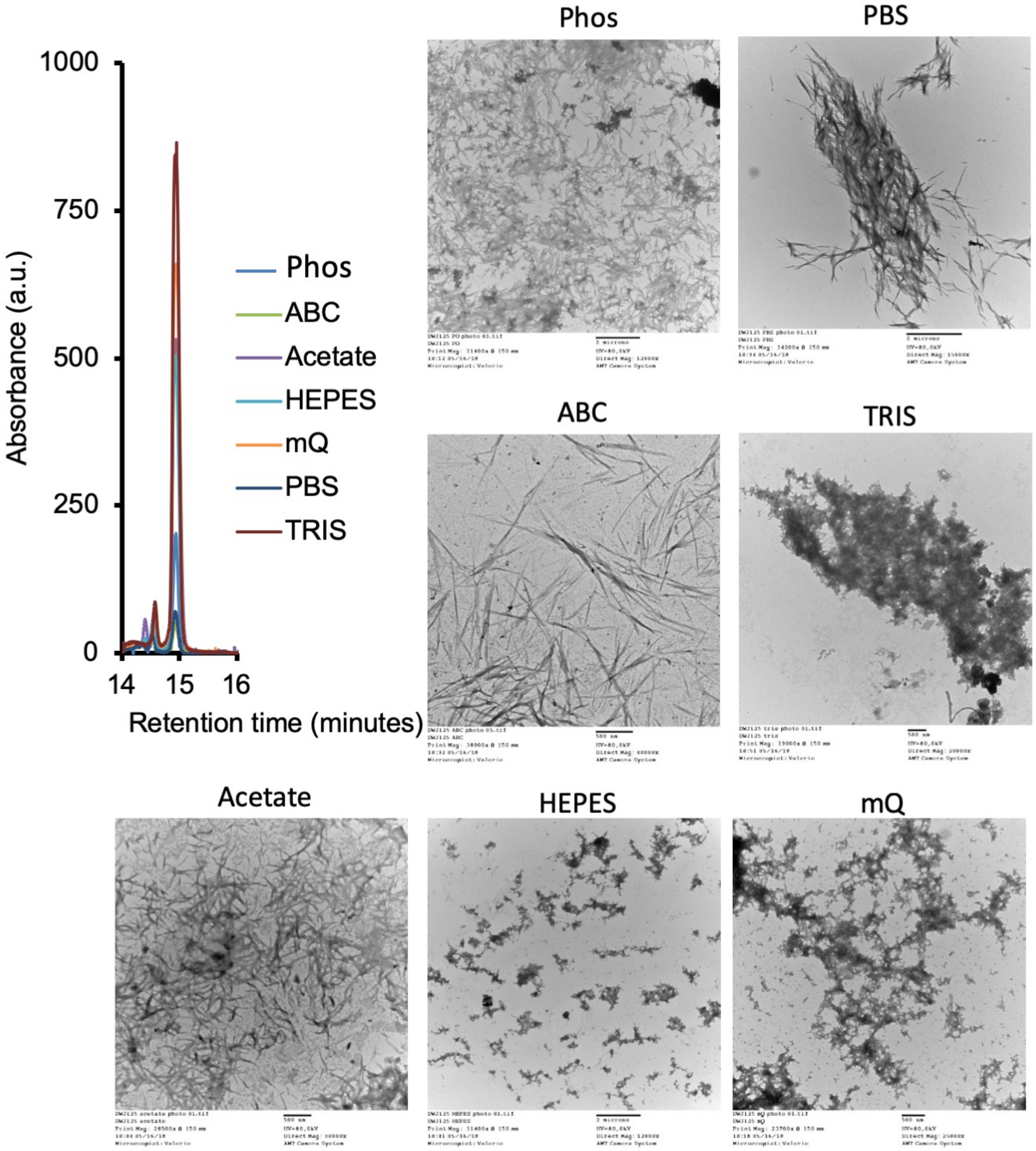

Figure S2. HPLC traces and EM pictures of the PGLa fibrillations described in Figure 3A.
